# Current Progress of Platelet-Rich Derivatives in Cartilage and Joint Repairs

**DOI:** 10.3390/ijms241612608

**Published:** 2023-08-09

**Authors:** Meng-Yi Bai, Vu Pham Thao Vy, Sung-Ling Tang, Truong Nguyen Khanh Hung, Ching-Wei Wang, Jui-Yuan Liang, Chin-Chean Wong, Wing P. Chan

**Affiliations:** 1 Graduate Institute of Biomedical Engineering, National Taiwan University of Science and Technology, Taipei 10607, Taiwan; 2 Adjunct Appointment to the Department of Biomedical Engineering, National Defense Medical Center, Taipei 11490, Taiwan; 3 International Ph.D. Program in Medicine, College of Medicine, Taipei Medical University, Taipei 11031, Taiwan; d142111018@tmu.edu.tw; 4 Department of Radiology, Thai Nguyen National Hospital, Thai Nguyen 24000, Vietnam; 5 Department of Pharmacy Practice, Tri-Service General Hospital, Taipei 11490, Taiwan; 6 Department of Orthopedics and Trauma, Cho Ray Hospital, Ho Chi Minh 70000, Vietnam; drhung.bvcr@gmail.com; 7 Department of Radiology, School of Medicine, College of Medicine, Taipei Medical University, Taipei 11031, Taiwan; 8 Department of Orthopedics, Shuang Ho Hospital, Taipei Medical University, New Taipei City 23561, Taiwan; chincheanwong@gmail.com; 9 Department of Orthopedics, School of Medicine, College of Medicine, Taipei Medical University, Taipei 11011, Taiwan; 10 International Ph.D. Program for Cell Therapy and Regenerative Medicine, College of Medicine, Taipei Medical University, Taipei 11011, Taiwan; 11 Department of Radiology, Wan Fang Hospital, Taipei Medical University, Taipei 116081, Taiwan

**Keywords:** platelet-rich fibrin, 3D PRF microstructure, cartilage, cytokines, growth factors, platelet-rich plasma

## Abstract

In recent years, several types of platelet concentrates have been investigated and applied in many fields, particularly in the musculoskeletal system. Platelet-rich fibrin (PRF) is an autologous biomaterial, a second-generation platelet concentrate containing platelets and growth factors in the form of fibrin membranes prepared from the blood of patients without additives. During tissue regeneration, platelet concentrates contain a higher percentage of leukocytes and a flexible fibrin net as a scaffold to improve cell migration in angiogenic, osteogenic, and antibacterial capacities during tissue regeneration. PRF enables the release of molecules over a longer period, which promotes tissue healing and regeneration. The potential of PRF to simulate the physiology and immunology of wound healing is also due to the high concentrations of released growth factors and anti-inflammatory cytokines that stimulate vessel formation, cell proliferation, and differentiation. These products have been used safely in clinical applications because of their autologous origin and minimally invasive nature. We focused on a narrative review of PRF therapy and its effects on musculoskeletal, oral, and maxillofacial surgeries and dermatology. We explored the components leading to the biological activity and the published preclinical and clinical research that supports its application in musculoskeletal therapy. The research generally supports the use of PRF as an adjuvant for various chronic muscle, cartilage, and tendon injuries. Further clinical trials are needed to prove the benefits of utilizing the potential of PRF.

## 1. Introduction

Platelet concentrates, also known as platelet-rich plasma (PRP) or platelet-rich concentrates (PRCs), have a history of several decades. Platelet concentrates were first produced in the early 1970s [1]. Since then, they have been used in various medicinal applications. They contain platelets, fibrin, growth factors, and mitogenic factors that can release cytokines, chemokines, and other factors. Hence, they can stimulate vessel formation, cell proliferation, and differentiation; improve angiogenesis; and decrease the occurrence of inflammation [2,3,4]. In the early 2000s, an evolution of PRP called platelet-rich fibrin (PRF) was introduced by Choukroun et al. [5], who prepared PRF by centrifuging blood without anticoagulants, which resulted in a fibrin matrix enriched with platelets and other blood components. PRF has gained popularity owing to its simplified preparation process and absence of biochemical modifications. PRF also contains many types of growth factors such as platelet-derived growth factor (PDGF- a/b and c), vascular endothelial growth factor (VEGF), hepatocyte growth factor (HGF), epidermal growth factor (EGF), connective tissue growth factor (CTGF), fibroblast growth factor (FGF), insulin-like growth factor (IGF), and transforming growth factor β1 (TGF-β1) [6,7,8]. Growth factors are transformed into active stages after platelet activation. They are mainly present in the α-granules of platelets [9] and have many functions, such as mediating cell proliferation and growth, angiogenesis, wound healing, and bone metabolism. Many PRF formulations of PRF been investigated, including leukocyte platelet-rich fibrin (L-PRF) [10,11], advanced platelet-rich fibrin (A-PRF) [10,12], titanium-prepared platelet-rich fibrin (T-PRF) [13,14], and injectable platelet-rich fibrin (i-PRF) [15,16]. The therapeutic potential of PRFs in regenerative medicine, especially in musculoskeletal regeneration [17], means they have promising clinical applications. In addition, many studies have reported that PRF has a positive effect not only on musculoskeletal injury but also in other fields, such as diabetic ulcers [18], cosmetic surgery [19], maxillofacial surgery [20,21], and cardiac surgery [22].

Despite the wide range of PRF applications, there is some variation in the outcomes when using PRF treatments. The present article aims to summarize the current relevant application of PRFs in the musculoskeletal system and provide the essential roles of components of PRFs in the musculoskeletal system and maxillofacial surgery.

## 2. Evolution of Platelet-Rich Derivatives

### 2.1. Fibrin Glue

Fibrin glue, also known as fibrin sealant, fibrin adhesive, or fibrin sutures, is the oldest and has been the most widely used fibrin clot for the past 40 years [1]. Fibrin glue has been used to mimic the blood clot mechanism. It was originally constituted by polymerizing fibrinogen, which is obtained from human plasma, along with thrombin and calcium. However, the fibrinogen concentration in plasma is very low (0.2% volume of whole blood) [23], so it is very difficult to obtain with autologous techniques and always results in unstable quality of the fibrin glue [24]. In addition, the non-industrial preparation of fibrin glue requires massive amounts of blood, time, and preparation to collect and obtain fibrinogen [25]. Therefore, autologous protocols are unsuitable for patients who require immediate surgery. 

Despite the advantages of autologous products, commercial fibrin glue has become the most popular fibrin glue since it became available on the market in 1998. Pharmaceutical companies such as Tisseel from Baxter Healthcare produce commercial fibrin glue as a surgical additive [26]. It is produced and supplied in separate vials with a dual syringe delivery system, in which human-plasma-derived thrombin containing ionic calcium and a fibrinogen-containing anticoagulant are first separated into two vials. Before use, these two components are immediately mixed using a syringe, and it is then ready for treatment. Although it has been claimed to be virus-inactivated, it still poses an infinitely low risk of viral contamination. Furthermore, because commercial fibrin glue is not an autologous blood-derived product, the risks of immune rejection, disease transmission, and manufacturing costs are also the main concerns for the market [27,28].

In a recent meta-analyses, fibrin glue was found to be effectively combined with polyglycolic acid sheets to reduce the risk of bleeding after endoscopic submucosal dissection for gastric cancer [29] and glue-based mesh fixation to reduce the incidence of chronic postoperative inguinal pain and hematoma after laparoscopic inguinal hernia repair [30]. However, the fibrin patch applied to the pancreatic stump did not reduce the incidence of postoperative pancreatic fistulas after distal pancreatectomy [31]. 

Fibrin glue embedded with drugs, antibiotics, cytostatic agents, or stem cells may increase the effectiveness of therapy and facilitate the targeted delivery of active substances for localized drug release. The use of fibrin glue alone resulted in suture repair for nerve regeneration [32]. There is clear evidence that fibrin glue combined with mesenchymal stem cells (MSCs) can regenerate nervous system lesions [33]. Articular cartilage repair using fibrin glue alone has not been reported. 

### 2.2. Platelet-Rich Plasma (PRP)

With the development of technology, scientists have been able to produce autologous fibrin glue (PPP) using a more simplified method [24,34], since some scientists have identified the important role of growth factors in cellular process regulation [35], while the focus of autologous fibrin glue research has been on new biomaterials named platelet concentrates [36]. Scientists have developed a first-generation platelet concentrate, PRP, as an autologous modification of fibrin glue [2,34]. PRP, a true concentrate of platelets, is a fibrin clot rich in platelets (containing 95% platelets, 4% red blood cells, and 1% white blood cells) that is obtained from plasma [36]. PRP is a safe and autologous product that is readily available at the point of care and minimizes the risks of immune rejection and disease transmission [37]. The PRP protocols have varied among researchers, although the main concept of the protocols remains consistent. They all include the concept of blood cell separation, regardless of the use of a typical centrifugation method or a commercial PRP kit called a cell separator [38]. They require whole blood to be separated by centrifugation into different layers of blood cells, red blood cells (RBC), buffy coat (leukocytes), and plasma. The basic rules of PRP protocols include three steps. First, an anticoagulant is added to whole blood before blood processing to stop blood coagulation. Second, the whole blood is processed using a two-step discontinuous centrifugation process or one-step centrifugation with a cell separator kit to obtain the preliminary PRP product. Third, after liquid PRP is obtained using various blood separation methods, with the addition of thrombin to activate fibrinogen and calcium chloride to neutralize the effect of anticoagulants, the fibrinogen in liquid PRP can finally be cross-linked and polymerized to form a gel-like fibrin clot.

Based on the biochemical compositions of PRP types resulting from different protocols, PRPs can be categorized into two groups: leukocyte-rich (L-PRP) and leukocyte-poor (P-PRP) [28]. Despite the differences between the compositions and protocols of all PRP types, they share common weaknesses. These include long processing times, high costs, poor mechanical properties [39], unstable product volumes, low fibrin densities, and weak fibrin polymerization [28], as well as the potential risk of allergic reactions and cross-contamination with bovine thrombin. PRP has limited potential to stimulate bone regeneration, as it releases growth factors quickly just before cell outgrowth from the surrounding tissue [40]. Therefore, in 2000, French scientists developed a new generation of platelet concentrate, PRF, to overcome the existing risks of PRP [5].

### 2.3. Platelet-Rich Fibrin (PRF)

For the purposes of this review, the PRF mentioned in this article is defined as a second-generation platelet concentrate, which was first developed by Choukroun et al. [5] and is also referred to as Choukroun’s platelet-rich fibrin [41] or leukocyte- and platelet-rich fibrin (L-PRF) [28] to avoid confusion with other PRF products that contain biochemical additions [42,43]. PRF was first developed in France for specific use in oral and maxillofacial surgery to avoid legal restrictions on the reimplantation of blood-derived products [24]. Unlike other platelet concentrates, fibrin glue, and PRP, PRF is not a blood-derived product; it is simply centrifuged without any biochemical blood handling.

#### 2.3.1. PRF Preparation

**Figure 1:**
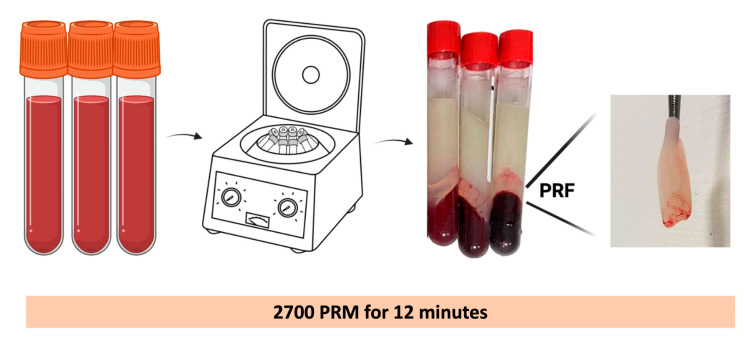
Illustration demonstrating the centrifugation of whole blood to obtain PRF. Created with BioRender.com.

PRF is an autologous platelet concentrate that is produced via one simple, short, and soft centrifugation cycle of 100% natural and non-additive whole blood. The first protocol for PRF production involved collecting 10 mL of a blood sample without anticoagulant, which is immediately centrifuged at 2700 rpm (approximately 400× *g*) for 12 min (Figure 1). In the absence of an anticoagulant, most platelets in a blood sample become activated within a few minutes of contact with the walls of the collection tube. This activation triggers the release of coagulation cascades, leading to blood clot formation. Initially, the fibrinogen is concentrated in the upper section of the tube. Subsequently, circulating thrombin interacts with fibrinogen, resulting in its conversion to fibrin. As a result, a fibrin clot is generated in the middle of the tube, positioned between the settled red blood cells at the bottom and acellular plasma at the top. The slow polymerization of fibrin during PRF preparation generates a fibrin clot with a fibrin structure that is very similar to that of natural fibrin. Comparing the fibrin structures of PRP and PRF, PRF seems to be a more friendly environment for cell migration and proliferation, and consequently cicatrization. The speed and method of the polymerization affect the 3D structure of the fibrin network. Fibrin glue and PRP favor the formation of bilateral fibrin glue owing to the high thrombin concentration and rapid polymerization. In contrast, PRF has a low thrombin concentration and slow polymerization rate, implying a more equilateral junction. Compared to the bilateral junction, the equilateral junction is a finer and more flexible fibrin structure with greater elasticity that provides better support for cytokine enmeshment and cellular migration [7,24]. 

Unlike fibrin glue and PRP, PRF is an autologous fibrin clot that requires only a small amount of money and time for production. PRF preparation is a simpler and more cost-effective process than PRP preparation [8,27,40,44] (Table 1). As PRF is an autologous platelet concentrate without any biochemical addition, it can avoid the risks of cross-infection, contamination, and product safety, which are the biggest concerns for commercial fibrin glues. PRF has several advantages over autologous fibrin glue. This method does not require a massive amount of blood for production and requires only 4 mL of fresh blood to produce 1 mL of PRF [45].

In contrast to PRP, PRF is an economical biomaterial and safe bio-scaffold. It is produced without the addition of anticoagulants or bovine thrombin, making it free from the risks of cross-contamination and immune rejection caused by biochemical adjuvants. The characteristics of fibrin glue, PRP, and PRF are summarized in Table 2. 

#### 2.3.2. Components of PRF

**Figure 2:**
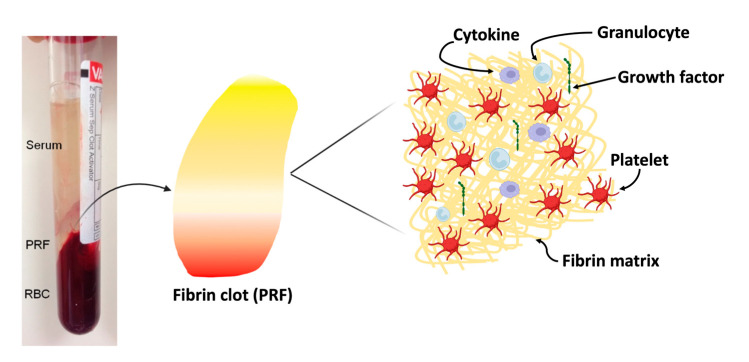
Components of PRF. Created with BioRender.com.

PRF is distinguished by its fibrin matrix, which contains a concentrated number of platelets, growth factors, cytokines, and other bioactive molecules (Figure 2). Platelets, the major components of PRF, are the primary cells responsible for the biological activity of PRF. Platelets are multifunctional cells that not only contribute to clotting but also have a significant impact on immune responses, wound healing, and tissue regeneration. These are stored by three types of particles (alpha, delta, and lambda) located inside platelets. Alpha granules are the most abundant and contain fibrinogen, immunoglobulins, and growth factors [54]. These granules contain various growth factors that are released through exocytosis when platelets are activated and are responsible for regenerating both soft and hard tissues after injury [55]. Delta granules contain adenosine diphosphate, tissue plasminogen activator, serotonin, and fewer lambda granules containing lysosomal enzymes [56,57]. PRF contains some of the most important growth factors, including platelets, cytokines, and granulocytes, as shown in Figure 2. 

PDGF is the initial growth factor present at the site of the injury. Platelet-released PDGF promotes the movement, multiplication, and survival of mesenchymal cells [7]. PDGF possesses chemotactic properties that attract macrophages to the site of injury. The combination of PDGF, TGF-β, and IGF produces a synergistic effect that stimulates blood vessel growth, facilitates cell division, promotes skin and bone matrix formation, and enhances collagen synthesis. With approximately 1200 PDGF molecules per platelet, the high concentration of PDGF in PRF may have a significant impact on wound healing and bone regeneration [8]. TGF-β is recognized as a factor that attracts macrophages, stimulates endogenous cells to secrete cytokines, and enhances the synthesis of the extracellular matrix, especially collagen I. Activated platelets secrete the active form of TGF-β1, which plays a significant role in stimulating fibroblast chemotaxis and promoting fibronectin and collagen production. It also acts as a protective factor by preventing collagen breakdown. Furthermore, TGF-β1 induces the formation of new blood vessels and attracts immune cells through chemotaxis [58]. Additionally, TGF-β1 enhances the proliferation and deposition of osteoblasts and inhibits the formation of osteoclasts and bone degeneration [8]. IGF exerts multiple effects on mesenchymal cells. It promotes differentiation and mitogenesis and stimulates growth and development [59]. Moreover, IGF-1 provides survival signals that protect cells from various triggers that induce apoptosis [7]. Additionally, IGF-1 stimulates chemotaxis, attracting and activating osteoblasts, which ultimately leads to increased bone formation [60]. IGF-1 also cooperates with growth factor TGF-β and bone morphogenetic proteins (BMPs) to participate in the synthesis of the basic substance of articular cartilage. EGF is an epidermal growth factor that promotes cell growth, differentiation, angiogenesis, and collagen formation [60]. Similarly, EGF promotes the secretion of cytokines by epithelial and mesenchymal cells [61]. EGF enhances the production and release of cytokines, which are important signaling molecules involved in various cellular activities, including immune responses, inflammation, and tissue repair.

In addition, PRF includes immune cytokines (Figure 2), such as interleukin (IL)-1β, IL-6, IL-4, and tumor necrosis factor (TNF)-α [24]. IL-1 is produced by most nucleated cells, such as monocytes, macrophages, B cells, NK cells, astrocytes, fibroblasts, endothelial cells, and neutrophils [62]. IL-1 has two subtypes, IL-1α and IL-1β. This particular cytokine enhances the production of adhesive molecules on endothelial cells and promotes the movement of phagocytes and lymphocytes towards the injury site [63]. In addition, they stimulate the helper T cells. IL-1β, in conjunction with TNF-α, triggers osteoclast activation and inhibits bone formation [64]. IL-6 is produced by T cells and macrophages to stimulate the immune response [65]. IL-6 is also produced by the muscle tissue and increases in response to muscle contraction. In contrast, IL-6 derived from osteoblasts stimulates osteoclasts, which in turn stimulates bone marrow cell destruction. This promotes the differentiation of B cells (white blood cells that produce antibodies), promotes growth in some cells, and inhibits growth in others. Moreover, IL-6 is an essential cytokine required to induce the transformation of naive T cells into cytotoxic T lymphocytes [64]. It is extensively produced during processes such as inflammation and tissue remodeling [66]. IL-4 is produced by macrophages and Th2 cells. It stimulates the proliferation of Th2 cells and promotes their differentiation into Th2 cells, which induces antibody-producing responses. It also stimulates the B cell class to convert IgE [67]. IL-4 can stimulate the activation of macrophages into M2 macrophages. The induced production of M2 macrophages leads to increased secretion of IL-10 and TGF-β, which reduces the severity of pathological inflammation. The increased secretion of M2 macrophages is closely associated with wound healing and fibrosis development [68]. TNF-α is a cytokine produced by various immune cells, primarily by macrophages. It plays a crucial role in inflammation, the immune response, and the regulation of cell death (apoptosis). TNF-α is involved in the recruitment and activation of immune cells such as neutrophils and monocytes to sites of infection or injury [69]. It induces the expression of adhesion molecules in endothelial cells, thereby facilitating leukocyte migration. TNF-α also stimulates the production of other inflammatory cytokines and chemokines, thereby amplifying the immune response [64]. Bai et al. [70] reported that the microstructure and proportion of PRF in a rabbit model were positively correlated with cytokine concentrations. PRF gel exhibited a quasi-graded distribution of PDGF-BB and TGF-β1. The concentrations of these cytokines in the PRF gel were significantly higher than those in the plasma because of the combination of two factors: (1) an extrinsic factor attributed to the fibrin gel structure; (2) the molecular characteristics of the different cytokines, which serve as intrinsic factors. Although cytokines are typically soluble and are expected to concentrate in the plasma after centrifugation, the highest concentrations of these cytokines were found at the red blood cell end of the gel. This suggests that the cytokines were stoichiometrically trapped in the PRF gel. They also analyzed the histology of PRF sections obtained from young and middle-aged men and women and showed a gradual increase in average porosity over time [71]. Furthermore, a decline in compactness was observed along the longitudinal axis of the PRF gel. They concluded that the section of the PRF gel nearest to the red blood cell layer is considered the core of the PRF clot and that both sex and age in humans influence their platelet generation capacity.

**Figure 3:**
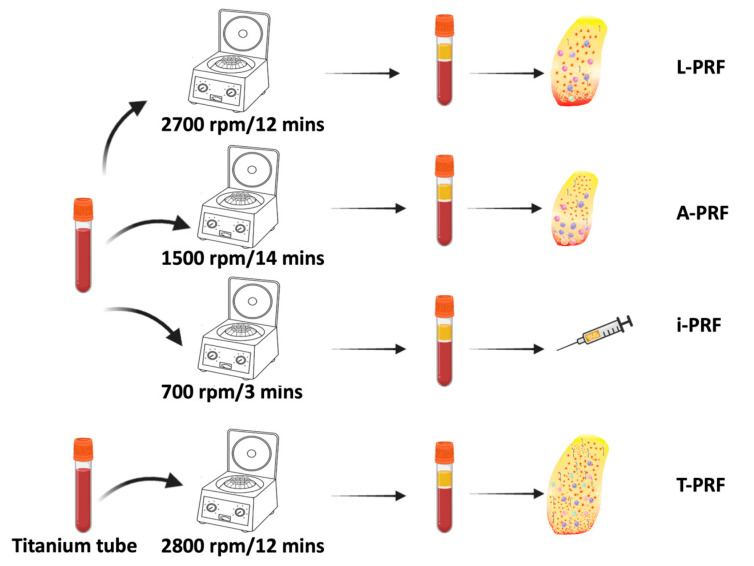
Various forms of PRF. A-PRF is synthesized from blood spun at 1500 rpm for 14 min leading leucocytes to shift to the bottom of the tube; i-PRF forms a platelet-rich yellow layer at the top and is easy to use in an injectable form; T-PRF is generated by using a sample of blood with titanium tube to centrifuge. T-PRF’s fibrin meshwork shows more firmness, thickness, and intricacy, thereby enhancing its overall consistency and integrity [14,72]. Created with Biorender.com.

Nevertheless, in recent years, there have been various alterations to the PRF protocol, resulting in the emergence of distinct products with diverse capabilities and potential uses in biology. Many formulations of PRF been investigated, including leukocyte platelet-rich fibrin (L-PRF), advanced platelet-rich fibrin (A-PRF), titanium-prepared platelet-rich fibrin (T-PRF), and injectable platelet-rich fibrin (i-PRF) (Figure 3).

In our previous studies, we investigated the characteristics of PRF and its clinical applications. Some novel observations from our series were reported for the first time, as follows: (1) the RBC portion of a PRF gel is characterized by the highest concentration of platelets and cytokines, making it an essential component known as platelet-rich fibrin essence (ePRF) [70]; (2) the reconstruction of the PRF microstructure in three dimensions from a series of two-dimensional SEM images revealing a dense fibrin matrix with a gauze-like surface morphology [70]; (3) the utilization of PRF and cartilage granules in the absence of bovine thrombin offers the possibility for a one-step cartilage repair surgery, which may yield favorable outcomes [45]. In rabbit models, we investigated the effectiveness of PRF in promoting meniscal tissue healing [73]. PRF stimulates cellular migration and enhances proliferation and ECM synthesis in cultured meniscocytes. Additionally, PRF contributed to increases in the formation and deposition of the cartilaginous matrix produced by cultured meniscocytes. We also developed a feasible one-step procedure to combine PRF and autologous cartilage grafts for articular chondral defects [74]. PRF has beneficial effects on the viability, differentiation, and migration of chondrocytes, and is a promising approach for cartilage repair [75]. Together, these data demonstrate the effectiveness of a single-stage, culture-free procedure combining the PRF and cartilage repair via cartilage autografts. In addition, we explored whether the ability to produce platelets from ePRF was influenced by human sex and age [71].

## 3. Clinical Application

### 3.1. Oral and Maxillofacial Surgery

Ghoneim et al. [76] evaluated the effectiveness of injecting injectable platelet-rich fibrin (i-PRF) into the joint space after arthrocentesis compared with arthrocentesis alone for treating patients with temporomandibular joint (TMJ) disc displacement with reduction. The findings revealed statistically significant decreases in pain intensity and clicking sounds, as well as increases in mouth opening and lateral movement, in the group of patients treated with i-PRF compared with the group treated with arthrocentesis alone. The TMJ has a distinct structure characterized by articular surfaces that are covered by nearly acellular fibrocartilage consisting of a minimal number of chondrocyte-like and fibroblast-like cells. Additionally, the predominant collagen type present in the TMJ is type I collagen, which is primarily synthesized by fibroblast-like cells. Kütük et al. [77] applied PRP to the right joints of rabbits and physiological saline to the left joints. The study demonstrated a significant increase in new bone regeneration in the PRP group compared with that in the saline group. A scanning electron microscopy analysis showed an improved ultrastructural architecture of the collagen fibrils, specifically in the PRP group. Giacomello et al. [78] assessed the efficacy of platelet-rich growth factor–Endoret^®^ injections for the treatment of TMJ osteoarthritis in 52 patients who were followed for 1 year. Their findings showed the effectiveness of platelet-rich in growth factor–Endoret injections in decreasing osteoarthritis symptoms and improving them over time.

### 3.2. Musculoskeletal Disorders

#### 3.2.1. Repair and Regeneration of Cartilage

In the last decade, platelet concentrates (such as PRP and PRF) have been widely investigated as useful therapeutic agents for the treatment of musculoskeletal disorders. PRF has been investigated as a potential treatment option for cartilage damage. The specialized connective tissue of diarthrodial joints is known as the articular cartilage. It is deficient in blood vessels, lymphatics, and nerves, leading to a limited natural ability to heal and repair itself. Owing to the absence of blood vessels in articular cartilage, it cannot trigger the same healing process as other tissues that have robust regenerative potential. However, the introduction of a PRF scaffold may simulate the initial stages of wound healing and tissue repair. The growth factors and cytokines present in PRF have been shown to exert chondrogenic and anti-inflammatory effects by enhancing the viability, differentiation, and migration of chondrocytes, which can support cartilage healing [75]. Since cartilage repair and regeneration are always limited by vascular inadequacy, it is important to have adequate nutrition in the development of cell-based therapies. PRF has been effective in providing nutritional support and increasing the number of cultured chondrocytes, comparable with other PRP-related in vitro studies [75]. In addition, PRF has been proven to provide an appropriate environment for the proliferation and maturation of chondrocytes; therefore, it can be used as a potential bioactive scaffold for cartilage regeneration [79]. Wong et al. [75] developed a one-stage method to combine PRF and autologous cartilage autografts for porcine articular cartilage repair. They reported that the regenerated cartilage surfaces in the treatment groups were smooth and continuous, suggesting that the cartilage repair was relatively complete. Chien et al. [80] demonstrated that human platelet-rich fibrin exudates with high platelet cytokine and growth factor levels can be integrated into biodegradable fibrin scaffolds for use as a regeneration matrix to stimulate chondrocyte proliferation and redifferentiation. In an injured cartilage rabbit model, Kuo et al. [45] reported the regenerative potential of cartilage with higher T2 values via MRI in the PRF-treated group compared to the control group, showing a reduction in proteoglycans and a progressive increase in collagen content. In a cartilage defect rabbit model, Taufik et al. [81] developed a treatment method involving the integration of microfractures, synovial grafts, and a PRF membrane. This combined method effectively promoted the regeneration of cartilage defects. The PRF membrane contributed essential growth factors, whereas the synovium supplied stem cells. The researchers observed significantly increased levels of aggrecan and type 2 collagen expression in the healing tissue of cartilage treated with microfracture and synovium–PRF transplantation. Several studies [82,83,84,85,86] have focused on the ability of chondrocytes to proliferate and differentiate in response to PRP and i-PRF. Appropriate cell types and chondrocytes are required for the first stage of cartilage tissue healing. Mustafa et al. [84] demonstrated that compared to PRP, the injection of i-PRF using the concept of low-speed centrifugation led to significantly increased chondrocyte activity and enhanced cartilage regeneration. Wang et al. [87] investigated the effects of arthroscopic surgery combined with PRP and PRF gels in 28 patients with defective knee cartilages. They showed that this combination therapy could repair knee cartilage defects, improve patient function, and relieve symptoms. Knee osteoarthritis (OA) is a chronic joint disease that mainly results from wear and tear and a progressive loss of articular cartilage. Many previous studies [88,89,90,91] have proven the positive effects of PRP or its combination with hyaluronic acid in the treatment of knee osteoarthritis, although few studies have investigated the benefits of PRF. Cheeva-Akrapan et al. [92] conducted a 36-month survival analysis of treatment with PRP enhanced with injectable PRF in osteoarthritis knee patients, resulting in an 80.18% survival rate in patients who did not require surgical intervention during the follow-up period. PRP releases growth factors shortly after injection, whereas PRF acts as a natural mesh for PRP and releases growth factors slowly.

#### 3.2.2. Meniscal Repair

Many meniscal injuries to the knee are caused by trauma, which results in instability and loss of joint function. They can lead to pain or disability, degenerative joint changes, and symptomatic osteoarthritis [93,94]. When the meniscus is damaged, especially because of the complete or partial cutting of the meniscus, it changes the distribution of forces on the meniscus, accelerating the process of knee osteoarthritis. Unlike PRP, which requires an additional scaffold for in situ tissue transplantation, PRF is a strictly autogenous fibrin-based biomaterial that encourages microvascularization and enables the local and progressive delivery of growth factors, which can be used to enhance bone and tissue regeneration [7,64]. Wong et al. [75] reported the positive stimulatory effects of PRF on meniscocyte migration, proliferation, and extracellular matrix synthesis in a rabbit model. Furthermore, they observed that PRF supplementation resulted in the increased formation and deposition of the cartilaginous matrix produced by cultured meniscocytes. Through morphological and histological evaluations, this study demonstrated that PRF facilitates meniscal repair in rabbits. Researchers have highlighted the potential benefits of using PRF to enhance the healing process of meniscal injuries. Narita et al. [95] employed biodegradable gelatin hydrogel as a carrier for PRP application in horizontal meniscal tears. They discovered that the combination of fibroblast growth factor-2 (FGF-2) with a gelatin hydrogel significantly promoted the proliferation of meniscal cells. Moreover, this combination effectively inhibited meniscal cell death for up to four weeks, leading to increased meniscal cell density and enhanced meniscal repair in a rabbit model. In contrast, PRP did not positively contribute to the healing process in Shin’s study [96]. They evaluated the effect of PRP on horizontal meniscal tears using an experimental rabbit model with a single injection. Griffin et al. [97] indicated that there was no significant difference in the clinical and functional scores between a group treated with PRP and a control group. Indeed, future prospective randomized studies with adequate sample sizes are needed to clarify the use of PRP and PRF in meniscal healing following meniscal repair. By conducting robust investigations, we can enhance our understanding of the role of platelets in this context and make informed decisions regarding their clinical application.

#### 3.2.3. Repair and Regeneration of Tendons

In addition, PRF has been reported to enhance tendon healing and improve clinical symptoms, particularly chronic pain. Owing to insufficient tissue vascularization, tendinopathies have a limited ability to repair and cause irreversible lesion symptoms. During tendon damage, PRF improves cellular and biomechanical responses and enhances the quality of repair. Alviti et al. [94] investigated the effectiveness of PRF in enhancing Achilles tendon healing and restoring tendon elongation through a gait analysis evaluation in 20 males during 6 months of follow-up compared with surgical repair alone. Controversially, Zumstein et al. [95] conducted a prospective randomized, controlled study of thirty-five patients randomized to receive arthroscopic rotator cuff repair with or without L-PRF at the repair site. Their findings revealed no significant improvement in structural integrity or tissue quality. In addition, the overall rates of non-healing were not significantly different between the two groups. Two systematic reviews and meta-analyses examining treatment with PRP reported that lateral epicondylitis, commonly known as tennis elbow, improves pain and function more effectively than corticosteroid injections in the intermediate term (12–26 weeks) [96] and for long-term follow-up (24 weeks post-treatment) [97]. Another systematic review demonstrated that PRP injection did not significantly reduce the pain intensity in chronic greater trochanteric pain syndrome compared with placebo injection (saline) [98], and a randomized, double-blind, controlled trial showed that PRP could achieve greater clinical improvements at 12 weeks than corticosteroid injection [99]. A systematic review of 34 randomized trials revealed that the use of PRP injections has a low risk of harm and is beneficial for long-term outcomes (≥12 months). Other systematic reviews of musculoskeletal disorders, such as carpal tunnel syndrome, patellar tendinopathy, and plantar fasciopathy, have described the promising efficacy of PRP treatment [100,101,102]. 

#### 3.2.4. Repair Ligament

The use of PRF for acute ligament injuries has also grown in popularity, despite the limited evidence. Matsunaga et al. [98] found that PRF scaffolds promote medial collateral ligament repair in rabbit models. In a rabbit model, Weng et al. [99] demonstrated the effectiveness of L-PRF in enhancing the biological healing of anterior cruciate ligament (ACL) mid-substance tears. When cultured in a three-dimensional environment, viable cells exhibited a high-density arrangement, forming layers on the surface of the L-PRF scaffold. This culture condition demonstrated significant cell ingrowth and the deposition of an abundant collagenous matrix. A comparative MRI study involved 44 patients with ACL injury who underwent arthroscopic ACL reconstruction with a semitendinosus tendon graft and intervention-sprayed PRF to the surface of the graft [100]. The results showed lower MRI signal intensities and less fluid in the tunnel in the PRF-treated group than in the control group. A study [101] evaluated the use of a platelet-rich plasma preparation rich in growth factors (PRGF) during ACL surgery, leading to more remodeling compared with the untreated graft group, with excellent ratings of 57.1% and 33.3%, respectively. Here, 77% of PRGF-treated grafts had histologically identifiable newly generated connective tissue enclosing them compared to 40% of the controls. Eggli et al. [102] reported that incorporating dynamic intraligamentary stabilization microfracturing and L-PRF led to stable clinical and radiological healing of torn ACL patients after one year. Given the existing in vitro and clinical evidence, further investigation is warranted to explore the role of PRF in tendon augmentation and repair. Specifically, a controlled randomized trial is necessary to assess its potential as a therapeutic modality for clinical use.

However, the outcomes of PRF administration in the treatment of musculoskeletal disorders are variable. For instance, two systematic reviews on the use of PRP injections in the treatment of rotator cuff tears reported that the constant shoulder scores, simple shoulder test scores, UCLA scores, and visual analog scale scores improved with PRP compared to the control group [103,104]. In contrast, PRF does not improve the tendon healing rates or functional outcomes [103]. The current evidence for the clinical application of PRP or PRF is summarized in Table 3. 

## 4. Conclusions and Future Perspectives

PRP and PRF continue to evolve as investigational treatments in maxillofacial surgery, musculoskeletal disorders, dermatology, and other fields. The inconsistencies in the clinical results were due to the large heterogeneity of the preparation protocols. Large variations in the centrifugal force and total centrifugation time significantly affected the platelet concentrations and growth factor release from the final products. The concentrations of PDGF-AB, TGF-β1, and VEGF in PRP were 3133–293,500, 20–153,863, and 0–44,000 pg/mL, respectively, according to the different centrifugation protocols [127]. Multiple modifications of basic protocols have led to the development of many techniques for obtaining PRP using either commercial centrifugation kits or manual or homemade procedures. Different protocols obtain different platelet concentrations; leukocyte concentrations; and growth factor (PDGF) concentrations (PDGF-AB, TGF-β1, and VEGF) according to Mariani et al. [127]. Similarly, the accumulated growth factor concentration ranges in PRF are 593-774 (VEGF), 23-36 (TGF-β1), and 859-1147 (EGF) pg/mL [128]. Furthermore, individual characteristics, especially age and sex, also influence the growth factor levels in PRF [71]. Therefore, a standard preparation protocol for PRP and PRF is necessary [129].

Our study obtained several results from the systematic reviews (Table 3). However, most systematic reviews concluded a lack of high-quality studies because the primary clinical studies included a small number of participants, were unblinded and unrandomized, lacked a proper control treatment, and lacked consistent treatment procedures for PRP or PRF. Although the standard treatment protocol for PRP and PRF is still being investigated, there is increasing evidence that PRP can provide positive outcomes in disease treatment. We suggest that more high-quality clinical trials are needed to determine the efficacy of PRF treatment and the most suitable treatment for PRP or PRF.

## Figures and Tables

**Table 1:** The advantages and disadvantages of platelet-rich fibrin.

Advantages of Platelet-Rich Fibrin	References
Preparation and application are easy and efficient	[46,47,48]
Natural biomaterial, obtained by autologous blood sample. No risk of infection, immune rejection, or a reaction	[5,47]
Does not require bovine thrombin and anticoagulants	[24,49]
Fibrin’s three-dimensional architecture with growth factors is better suited for tissue regeneration	[50]
It can increase the healing rate	[51,52]
Decreased patient bleeding, discomfort, and pain after surgery	[47]
**Disadvantages of Platelet-Rich Fibrin**	
Autologous blood means the final amount is limited	[53]
Short handling time and fast degradation	[40]

**Table 2:** Characteristics of fibrin glue, PRP, and PRF.

	Type		Process		Concentration		Fibrin	Safety and Risk	
	Name	Blood Source	Time	Cost	Platelet	Leukocyte	Density	Cross-Infection	Immune Rejection
Fibrin Glue	Tisseel	Commercial	Very Long	High	None	None	High	High	High
	PPP	Autologous	Very Long	Very High	None	None	Low	Low	Low
PRP	L-PRP	Autologous	Long	High	Low	High	Low	Low	Low
	P-PRP	Autologous	Long	High	High	None	Low	Low	Low
PRF	L-PRF	Autologous	Short	Very Low	High	Very High	High	None	None

**Table 3:** Current evidence of clinical applications of PRP and PRF.

Indication or Procedure	PRP	PRF
Achilles tendinopathy	PRP is not superior to placebo treatment [105]	
Acute muscle injuries	Abundance of high-quality evidence [106]	
Aging skin	Temporarily induce modest improvement in facial skin appearance, texture, and lines [107]	
Alopecia areata	Abundance of high-quality evidence [108]	Leukocyte PRF is superior to control treatment [109]
Androgenic alopecia	PRP is likely to reduce hair loss, increase hair diameter and density [110]	
Arthrogenous temporomandibular disorders	PRP is more effective than conservative treatments [111]	
Carpal tunnel syndrome	PRP represents a promising therapy for patients with mild to moderate CTS [112]	
Diabetic ulcers	PRP may improve ulcer healing [113]	
Elbow tendinopathy	PRP injections improved pain and function more effectively than corticosteroid injections at the long-term follow-up [114] PRP significantly improved pain and elbow function in the intermediate term (12–26 weeks) [115]	
Greater trochanteric pain syndrome	PRP is not superior to placebo treatment [116] A single PRP injection is superior to a single corticosteroid injection [117]	
Knee osteoarthritis	PRP injections have a low risk of harm and beneficial ≥12 month outcomes [118]	
Mandibular third molar surgery		Decrease in prevalence of alveolar osteitis [119]
Maxillary sinus augmentation		Improves the healing period and bone formation [120]
Medication-related osteonecrosis of the jaw	Abundance of high-quality evidence [121]	Abundance of high-quality evidence [121]
Patellar tendinopathy	Multiple injections of PRP obtained positive outcome [122] Pain relieving and functional improvement [123]	
Plantar fasciopathy	PRP may provide a long-term effect in relieving pain [124]	
Ridge preservation procedure		L-PRF reduced the magnitude of vertical and horizontal bone resorption [125]
Rotator cuff tears	PRP treatment decreases the retear rate and improves the clinical outcomes [104] PRP improves patient outcomes [103]	L-PRF yields no beneficial effect in clinical outcome [126] PRF has no benefit in improving patient outcomes [103]

## Data Availability

Data are contained within the manuscript.
